# The Effect of the Supplementation of Humic Substances and Fermented Products in the Feed on the Content of Salinomycin Residues in Poultry Tissues

**DOI:** 10.3390/foods13010068

**Published:** 2023-12-24

**Authors:** Simona Hriciková, Ivona Kožárová, Beáta Koréneková, Slavomír Marcinčák

**Affiliations:** Department of Food Hygiene, Technology and Safety, University of Veterinary Medicine and Pharmacy in Košice, Komenského 73, 04181 Košice, Slovakia; simona.hricikova@student.uvlf.sk (S.H.);

**Keywords:** salinomycin, ELISA, analysis, humic substances, fermented products

## Abstract

The presence of antimicrobial residues in products of animal origin is a constant problem for consumer health. The aim of this study was to observe the effect of the addition of humic substances (H), fermented products (F) and a mixture of both (FH) to feed supplemented with the coccidiostat salinomycin, compared with a control group (C), on the content of salinomycin residues in the edible tissues of broiler chickens using two microbial inhibition screening methods, Explorer 2.0 test and the Screening Test for Antibiotic Residues (STAR), and a confirmatory competitive enzyme immunoassay analysis (Salinomycin ELISA Kit). The results of the microbial inhibition tests showed a gradual decline in the positive results in the tissue samples from the last day of salinomycin administration (30th day) tothe last day of fattening (37th day, day of slaughter) in group C and no positive results in the tissue samples from experimental groups H, F and FH slaughtered on the last day of fattening. Using the Salinomycin ELISA Kit, salinomycin was detected in the chicken muscle tissues of all the control and experimental groups. However, no sample from any group contained salinomycin at a concentration exceeding the maximum residue limits set by European law. The high level of significance (*p* < 0.001) confirmed the positive influence of the administration of humic substances and fermented products on the content of salinomycin residues in chicken tissues.

## 1. Introduction

The monocarboxylic polyether acid ionophore salinomycin was initially identified by Miyazaki et al. from *Streptomyces albus* [1]. In the poultry industry, salinomycin as a coccidiostat was utilised as a feed additive for chickens for fattening and chickens reared for laying in accordance with Commission Implementing Regulation (EC) 2017/1914. Moreover, salinomycin demonstrates antimicrobial action, particularly in relation to Gram-positive bacteria. Antiviral, antifungal, antiparasitic, and anti-inflammatory activities and, most recently, initiatives to combat cancer are also mentioned in relation to salinomycin [2,3,4].

Coccidiostats are chemicals either obtained via synthesis or produced by micro- organisms, which inhibit or kill highly host-specific protozoan parasites of the genera *Eimeria*, which cause coccidiosis in farmed animals [5]. Even in the presence of high sanitary standards and good management, coccidiosis occurs, with a serious potential impact on animal health and welfare and with high mortality rates [6]. Aside from intensively farmed animals, such as poultry, coccidiosis also affects extensively reared species, including sheep, cattle, pigs and rabbits [7,8,9]. Due to the self-limiting nature of the life cycle and enhanced resistance to reinfection, coccidiosis is rarely a problem in extensively raised systems, althoughit becomes important in closely confined and highly intensive production systems [10].

Use of coccidiostats in modern poultry production represents the main method of controlling coccidiosis. Coccidiostats are administered to the feed at the authorised levels throughout the life of the animal (in the case of chickens for fattening) in order to protect against re-infection from the ever-present oocyst stage of the disease [5]. The occurrence of coccidiostats in feed may result in the presence of residues of these substances in food products of animal origin [11,12].

The presence of antimicrobial residues in products of animal origin is a constant problem for consumer health. Regulation (EU) 2017/625, in accordance with the relevant tertiary legislation, imposes an obligation to carry out official controls for residues of pharmacologically active substances in products of animal origin. In the production of poultry meat and poultry meat products, it is essential to control the presence of residues of all the coccidiostats classified according to Regulation (EC) 1831/2003 as feed additives used in poultry nutrition [12,13].

A screening technique is a way to find out if a chemical or class of compounds is present at the relevant level. The residue or a particular residue metabolite must be detectable via the procedure at or below the maximum residue limit [14]. The maximum residue limit (MRL) is the maximum allowed concentration of a residue in a food product obtained from an animal that has received a veterinary medicine or that has been exposed to a biocidal product for use in animal husbandry [4]. The European Medicines Agency’s (EMA) Committee for Veterinary Medicinal Products (CVMP) is responsible for recommending MRLs, which, when adopted by the European Commission, become legally binding food safety standards [15].

Rapid screening methods must be fast and reliable. Such methods include agar diffusion tests using a medium inoculated with a susceptible bacteria and rely on agar diffusion of the antibiotic residues, which is expressed by the formation of an inhibition zone based on the presence of antibiotic residue in the sample (plate tests),or based on the change in colour of the indicator conditioned by the growth of the test strain of bacteria present in the agar (tube tests) [2,16,17].

Since rapid screening tests usually evaluate the result of sample contamination only at the qualitative level (positive or negative) or semiquantitative (high, medium/low, or negative), the confirmation of the results is essential. To determine the type and concentration of an antibiotic in the contaminated sample, specific methods based on immunochemical or chromatographic techniques should be used [2,16,17].

Properties such as the speed and simplicity of tube tests offer the possibility of their use in various areas, such as processing plants, slaughterhouses or laboratories. Explorer 2.0, produced by Zeulab (Zaragoza, Spain), is one of the qualitative tests that can be used to identify inhibitory compounds in meat muscle, liver, kidney, egg, feed and blood. This test screens more than 50 antibiotics from various groups, aminoglycosides, macrolides, tetracyclines, beta-lactams, quinolones and sulphonamides, thus avoiding problems in the manufacture of fermented products, as well as helping to comply with the maximum residue limits established by the European Union. Spores of the test organism *Bacillus stearothermophilus* var. *calidolactis* present in the agar grows during incubation in the absence of antibiotic residues, as manifested by a colour change (yellow colour) of the pH indicator in the acidic environment produced by bacterium. In the presence of antimicrobial residues, the growth of the test organism will be inhibited, the pH of the agar will remain alkaline and the colour will remain purple [18,19]. The system has been validated and recommended by internal validation according to ISO 13969:2003 [20], external validation by the Spanish Agency for Food Safety and Nutrition (AESAN), and external validation by the Belgian Reference Laboratory ILVO, and the European Reference Laboratory, ANSES [20].

The Screening Test for Antibiotic Residues (STAR) developed by the Community Reference Laboratory AFFSA in Fougeres (France) is validated for antibiotic residue detection in milk [16] and in meat [21]. The STAR method is based on five test plates that are susceptible to the following groups of antibiotics: for aminoglycosides, *Bacillus subtilis* BGA (pH 8.0), for beta-lactams and macrolides, *Kocuriarhizophila* ATCC 9341 (pH 8.0), for tetracyclines, *Bacillus cereus* ATCC 11788 (pH 6.0), for quinolones, *Escherichia coli* ATCC 11303 (pH 8.0), and for beta-lactams and sulphonamides, *Bacillus stearothermophilus* var. *calidolactis* ATCC 10149 (pH 7.4). After incubation, the presence of an antibiotic is indicated by the production of an inhibition zone around the sample. The STAR method was intended to be the preferred method for residue screening because of its specificity and detection capacity concerning antibiotic residues throughout several European countries and is the officially approved protocol for the screening of products (meat, edible organs, milk, eggs) of food-producing animals for antibiotic residues in Slovakia [2,16,21,22].

Immunoassays are commonly used to measure antibodies, antigens, proteins and glycoproteinsin biological samples. Especially the Enzyme-Linked Immunosorbent Assay (ELISA) hasproved to be an efficient method for the analysis of numerous biological samples. It is a simple, rapid, sensitive, and high-throughput assay, which is the most widely used and the most time-efficient developing method of enzyme immunology. The ELISAis widely used in the biochemical and clinical fields and is rapidly becoming available for pesticides and veterinary drugs such as antibiotics for screening purposes [17,23]. The principle of the ELISA method relies on antibodies to detect a target antigen using highly specific antibody–antigen interactions, where the catalytic properties of enzymes to detect and quantify immunologic reactions are used. The anti-salinomycin monoclonal antibody was initially prepared by Muldoon et al. (1995), who developed a competitive inhibition ELISA for salinomycin residues. It was possible to detect salinomycin at levels as low as 50 ng·g^−1^ in chicken liver [24]. The Salinomycin ELISA Kit produced by Creative Diagnostics (New York, NY, USA) is a competitive colorimetric enzyme immunoassay for the quantitative analysis of salinomycin in meat, milk and animal feed. The drug coated on the plate interacts with the specific primary antibody, and a secondary enzyme-labelled antibody is subsequently bound to this complex. After addition of the substrate, the colour intensity read with a 450 nm wavelength has an inverse relationship with the target concentration in the sample [25].

Due to the extensive use of antimicrobial substances in the animal products industry and with the aim of innovation, several natural substances are offered, which by their nature can affect the content of antimicrobial substance residues in products of animal origin and thereby contribute to consumer protection. Due to the presence of functional groups in their molecules, humic substances can reduce the presence of residues of pharmacologically active substances in animal products [26,27,28]. By-products such as wheat bran are traditional feed ingredients. Although they contain multiple nutrients, including vitamins and antioxidants, the entrapment of these nutrients by hemicellulose xylan and other non-starch polysaccharides (NSPs) blocks digestion and intestinal absorption and affects the composition of gut microorganisms [29]. The fermentation process uses the ability of selected microorganisms to produce non-starch polysaccharides, which help degrade harmful polysaccharides in feed. This results in a more favourable balance between fermentable carbohydrates and protein, a ratio believed to be critical for maintaining good gut health [30,31]. Due to the chemical predispositions of humic substances in synergy with the effect of fermented products added to poultry feed, it is possible to assume a decrease in salinomycin residues in the investigated samples of poultry tissues.

Since the condition and health of the intestinal tract and intestinal microflora of animals play an important role in the absorption and effectiveness of drugs, and given that antimicrobial residues in products of animal origin is a constant problem for consumer health, the aim of this study was to observe the effect of the addition of humic substances and fermented products on the content of salinomycin residues in poultry tissues via available screening methods using bacterial strains sensitive to antimicrobial substances and confirmatory immunoassays.

## 2. Materials and Methods

### 2.1. Standards

Salinomycin (Sigma-Aldrich S4526 PtyLtd., Darmstadt, Germany), streptomycin (Sigma-Aldrich S6501), tylosin (Sigma-Aldrich T6134), chlortetracycline (Sigma-Aldrich C4881), ciprofloxacin (Sigma-Aldrich 17850), and sulphamethazine (Sigma-Aldrich S6256).

The concentrations of the antibiotic standard solutions were prepared as follows: salinomycin (50 µg·L^−1^, 100 µg·L^−1^, 50 µg·L^−1^), streptomycin (2000 µg·L^−1^), tylosin (1000 µg·L^−1^), chlortetracycline (200 µg·L^−1^), ciprofloxacin (100 µg·L^−1^), sulphamethazine (1000 µg·L^−1^). The preparation and storage of the standard solutions was in accordance with the procedure of the methods used.

### 2.2. Media and Strains

Antibiotic medium 11 (Difco 259310; Difco, Detroit, MI, USA), DST test agar pH 7.4 (CM 261; Oxoid, Basingstoke, UK), test agar pH 6.0 (Merck 10663; Merck, Darmstadt, Germany), and test agar pH 8.0 (Merck 10664; Merck, Darmstadt, Germany). The culture media were prepared according to the manufacturer’s instructions.

*Bacillus cereus* ATCC 11778 (Czech Collection of Microorganisms, Brno, Czech Republic), *Bacillus stearothermophilus* var. *calidolactis* ATCC 10149 (Merck 1.11499, Merck, Darmstadt, Germany), *Bacillus subtilis* BGA (Merck 10649), *Escherichia coli* ATCC 11303 (Czech Collection of Microorganisms) and *Kocuriarhizophila* ATCC 9341 (Czech Collection of Microorganisms). *Bacillus stearothermophilus* var. *calidolactis* (Explorer 2.0 kit, Zeulab, Zaragoza, Spain), ready to use. Freeze-dried bacterial strains of *Kocuriarhizophila* ATCC 9341, *Escherichia coli* ATCC 11303 and *Bacillus cereus* ATCC 11778 were revived in accordance with the supplier’s instructions. Bacterial suspensions of *Bacillus subtilis* BGA and *Bacillus stearothermophilus* var. *calidolactis* ATCC 10149 were prepared according to the manufacturer’s instructions.

### 2.3. Experimental Animals and Sample Collection

The Ethical Committee for Animal Care and Use of the University of Veterinary Medicine and Pharmacy in Košice (Slovak Republic) approved the animal protocol for this research as clinical trial EKVP/2022-09.

One hundred and sixty one-day-old broiler chickens (*Gallus*, meat hybrid COBB 500) were randomly evenly divided into four groups: three experimental groups (H, F and FH) and a control group (C). The chickens were fattened and placed on deep litter while ensuring and controlling the microclimate conditions. The humidity was monitored and maintained at about 70%. The temperature was monitored, with the initial temperature of 33 °C, which was reduced to 21 °C after afew days. The chickens were placed in animal care-approved cages with free access to feed and water and fed commercial feed BR1, BR2 and BR3 (De Heusa.s., Bučovice, Czech Republic), respectively. The chickens were fed with commercial feed BR1 containing nicarbazine at aconcentration of 101 mg·Kg^−1^ from the 1st to the 10th day of fattening. The feed BR2 was administered to the chickens from the 11th to the 30th day of fattening with salinomycin content at aconcentration of 70 mg·Kg^−1^. The chickens were fed with BR3 feed from the 31th day until 37th of fattening without any antimicrobial drug present. Supplements of the individual groups of chickens: (1) Experimental group H—experimental group H was fed commercial feed BR1, BR2 and BR3 supplemented with 0.7% of humic substances (HumacNatur AFM, Humacs.r.o., Košice, Slovakia) from the first day of fattening; (2) Experimental group F—chickens in experimental group F were fed commercial feed BR1, BR2 and BR3 supplemented with fermented products at aconcentration of 10% from the 11th day of fattening (fermented products consisted of 50% wheat bran fermented using the strain *Cunninghamella elegans* CCF2591 and 50% of corn feed fermented using the strain *Mortierellaalpina* CCF2861); (3) Experimental group FH—chickens in experimental group FH were fed commercial feed BR1, BR2 and BR3 supplemented with a combination of humic substances at aconcentration of 0.7% (from the first day of fattening) and fermented products at a concentration of 10% (from the 11th day of fattening); and (4) Control group C—chickens were fed commercial feed BR1, BR2 and BR3 without any supplements. The specific concentrations of the humic substances and fermented products were chosen based on previous studies [32,33,34,35,36,37,38].

The health state of the broiler chickens was checked daily. All the chickens were slaughtered in line with Council Regulation (EC) No. 1099/2009 [39] on the protection of animals at the time of killing by manual cervical dislocation followed by bleeding. After evisceration, meat and the respective edible organs were collected for further laboratory analysis. On the last day of fattening (37th day), two chickens from each experimental group (H, F and FH) were slaughtered and the respective samples of muscle (homogenised mixture of breast and thigh muscles), gizzard, heart, liver, spleen and kidney were withdrawn and stored at −20 °C until analysis. The chickens from the control group were slaughtered on the 30th day of fattening (last day of BR2 feed administration; samples C(30)), on the 33th day of fattening (in the middle of BR3 feed administration, samples C(33))and on the last day of fattening (37th day; samples C(37)). The respective samples of muscle (homogenised mixture of breast and thigh muscles), gizzard, heart, liver, spleen and kidney were withdrawn and stored at −20 °C until analysis.

### 2.4. Methods

Microbial inhibition tests: Explorer 2.0 (Zeulab, Zaragoza, Spain) and STAR [22].

Enzyme-Linked Immunosorbent Assay (ELISA): Salinomycin ELISA Kit (Creative Diagnostics, New York, NY, USA).

### 2.5. Determination of Sensitivity of Antibiotic Standards Using STAR and Explorer 2.0

Explorer 2.0: A total of 100 µL of salinomycin standards at the concentrations of 50 µg·L^−1^, 100 µg·L^−1^, and 50 µg·L^−1^ wasadded in different tubes, respectively.The tubes were sealed carefully with the adhesive film and placed in the e-Reader device (Zeulab, Zaragoza, Spain). The instructions for thee-Reader device were followed to begin the assay. After incubation at 65 °C ± 1 °C, the device stopped the assay automatically and the results were shown on screen. An absorbancethatrepresents positive or negative results (Explorer 2.0) was evaluated to determine the concentration of the respective salinomycin standards, which completely inhibits the growth of the test organism.

STAR: A total of 30 µL of streptomycin, tylosin, chlortetracycline, ciprofloxacin and sulphamethazine was pipetted onto two 9mm filter paper discs placed on the surface of the agar medium of the test plates, respectively. Incubation of the plates wasprovided as follows: *Bacillus cereus* ATCC 11788 and *Bacillus subtilis* BGA at 30 °C for 24 h, *Escherichia coli* ATCC 11303 and *Kocuriarhizophila* ATCC 9341 at 37 °C for 24 h, and *Bacillus stearothermophilus* var. *calidolactis* ATCC 10149 at 55 °C for 15 h. After incubation, the mean diameters of the inhibition zones (mm) with the expression of the standard deviation (±SD) were evaluated for each plate, respectively. Sterile demineralised water was used as a negative control (NC).

### 2.6. Pre-Preparation of Samples

Explorer 2.0: The meat muscle, liver and kidney samples were stored at −20 °C until analysis. The juice was prepared by heating approximately 3 ± 0.5 g (meat muscle) and 5 ± 0.5 g (liver and kidney) of the tissue in a heat-resistant tube dipped in a beaker of water and placed in a microwave on the“defrost” setting for 3–4 min. The released juice was collected into new tubes.

STAR: The samples of chicken tissues, muscle, stomach, heart, liver, spleen and kidney, were stored at −20 °C until analysis.

ELISA: All the reagents were prepared according to the manufacturer’s instructions. A total of 3 ± 0.05 g of homogenised muscle samples was loaded into a 50 mL centrifuge tube with 6 mL of methanol (67561 Merck, Darmstadt, Germany) and vortexed for 30 min. Consequently, the tubes were centrifuged at room temperature for 5 min at 4000× *g*. A total of 3 mL of clear supernatant was transferred to a new tube and added 1 mL of 2 M NaOH solution (1310732 Merck), 6 mL of N-hexane (110543 Merck) and vortexed for 1 min. After centrifugation for 5 min at 4000× *g*, 3 mL of clear supernatant was transferred to a new tube and dried under 55 °C water bath nitrogen-blowing (Turbo-Vap LV, Zymarck, Germany). The residue was diluted in 0.5 mL of Sample Dilute and vortexed for 10 s. The recovery range of the sample extraction method provided was 90% ± 30%.

### 2.7. Detection of Salinomycin Residues in Incurred Samples

Explorer 2.0: A total of 100 µL of negative control (Zeulab, Zaragoza, Spain) sample and 100 µL of sample (meat muscle, liver and kidney) were added in different tubes, separately. The test tubes were incubated at room temperature for 20 min. Sealing the tubes was not needed in this step. After incubation, the tubes were turned upside down to remove the remaining samples. The wells were washed by filling them with distilled water. The excess water was removed by turning the tubes upside down on top of an absorbent paper. The tubes were sealed carefully with the adhesive film and placed in the e-Reader device (Zeulab, Zaragoza, Spain). The instructions for thee-Reader device were followed to begin the assay. After incubation at 65 °C ± 1 °C, the device stopped the assay automatically and the results were shown on screen.

STAR: The 2 mm slices of the samples were obtained by cutting from an 8 mm × 2 cm cylindrical core removed from the frozen tissues using a cork borer. The slices were placed in parallel onto the surface of the agar medium in Petri dishes. The plates were incubated as follows: the plates seeded with *Bacillus cereus* ATCC 11788 and *Bacillus subtilis* BGA at 30 °C for 24 h, the plates seeded with *Escherichia coli* ATCC 11303 and *Kocuria rhizophila* ATCC 9341 at 37 °C for 24 h, and the plates seeded with *Bacillus stearothermophilus* var. *calidolactis* ATCC 10149 at 55 °C for 15 h.

ELISA: The required number of pre-coated strips were inserted into the ELISA rack and 50 µL of standards/samples was added in quadruplicate into different wells. The standards were added in the order from low concentration to high concentration. Consequently, 50 µL of Enzyme-Conjugated Antibody solution was added into each well. Afterwards, 50 µL of Salinomycin Antibody was added into each well and mixed well by gently rocking the plate manually for 30 s. The plate was incubated at room temperature for 30 min. After incubation, the wells were washed 4 times with 260 µL of 1 × Wash Solution, whereas after the last wash, the plate was inverted and gently tapped dry on absorbent paper. A freshly prepared mixture of Solution A and Solution B was added ata volume of 100 µL into each well and the plate was gently mixed via manual rocking for 1 min. The plate was incubated for 15 min at room temperature. After incubation, 50 µL of Stop Buffer was added into each well to stop the enzyme reaction. Immediately after adding the Stop Buffer, the plate was read on a plate reader with a 450 nm wavelength.

### 2.8. Reading the Test Results

Explorer 2.0: Explorer 2.0 is based on the inhibition of microbial growth. The kit is supplied in a single-tube format. Each tube contains agar medium spread with *Geobacillus* thermophile spores and a pH indicator. When the test is incubated at 65 °C, spores germinate and cells grow, producing acid and changing the agar pH. Variations in the pH will produce changes in the agar colour from purple to yellow. When samples contain inhibitors at higher concentrations than the limit of detection, the bacteria will not grow and neither colour change will be observed. The results were read automatically with an e-Reader (Zeulab, Zaragoza, Spain). The device detects the colour change inthe negative control via photometrical (595 and 650 nm) reading, determines the cut-off value and automatically terminates the incubation time of the samples with a positive sample expressed as a value ≥56 and a negative sample as a value <56. Yellow colour indicates the absence of salinomycin residues. Purple colour indicates the presence of salinomycin residues at or above the LOD of the test.

STAR: The principle of the method is the agar diffusion test, in which test strains are used on five Petri dishes with agar medium: *Bacillus subtilis* BGA, *Kocuria rhizophila* ATCC 9341, *Bacillus cereus* ATCC 11788, *Escherichia coli* ATCC 11303 and *Bacillus stearothermophilus* var. *calidolactis* ATCC 10149. The prepared samples are placed on the surface of the test agar medium with the test strains. Incubation, in which normal growth of the test strain occurs, causes turbidity of the agar medium. If substances inhibiting the growth of the test strain are present in the tested sample, clear zones appear around the sample. The size of the inhibition zones depends on the concentration and type of antimicrobial substance present in the tested sample. These zones are compared with the size of zones formed by control solutions of the antibiotics streptomycin, tylosin, chlortetracycline, ciprofloxacin and sulphamethazine. The samples are considered positive if they show a zone of inhibition greater than 2 mm on *Kocuria rhizophila* ATCC 9341, *Escherichia coli* ATCC 11303, *Bacillus subtilis* BGA and *Bacillus cereus* ATCC 11788 plates and/or a zone of inhibition greater than 4 mm on the *Bacillus stearothermophilus* var. *calidolactis* ATCC 10149 plates.

ELISA: The Salinomycin ELISA Kit (Creative Diagnostics, New York, NY, USA) is a competitive enzyme immunoassay for the quantitative analysis of salinomycin in meat, milk and animal feed. The kit is based on a competitive colorimetric ELISA assay. The drug of interest has been coated on the plate wells. During the analysis, the sample is added along with the primary antibody specific for the target drug. If the target is present in the sample, it will compete for the antibody, thereby preventing the antibody from binding to the drug attached to the well. The secondary antibody, tagged with enzyme, targets the primary antibody that is complexed to the drug coated on the plate wells. The resulting colour intensity, after the addition of the substrate, has an inverse relationship with the target concentration of the sample. Afterwards, the plate is read on a plate reader with a 450 nm wavelength. The obtained absorbance values of the standards were used to create a standard curve using the software Microsoft Office Excel 2019. The concentration values of the standards were taken as the X-coordinates, and the absorbance values were taken as the Y-coordinates. To connect each coordinate point, the trend line was created. Based on the trend line, the trend line equation was generated. The concentrations of salinomycin were calculated based on the obtained regression equation. The final concentrations of salinomycin in the examined samples were calculated according the created regression equation after substituting the measured absorbance values into the equation. Finally, the correlation coefficient was expressed.

### 2.9. Data and Statistical Analysis

Data from the STAR and ELISA methods were evaluated using the software Microsoft Office Excel 2019. The data concerning the sizes of the inhibition zones are presented as the mean ± standard deviation (SD) of six measures. The data concerning the absorbance measured at 450 nm are presented as the mean ± standard deviation (SD) of four measures.

Statistical analysis of the ELISA results data was performed using the statistical software GraphPad Prism 8.3.0.538 (GraphPad Software, San Diego, CA, USA). ANOVA and Tukey’s test for multiple comparisons of data means with a confidence interval set at 95% were performed. The statistical analysis was performed via one-way analysis of variance.

## 3. Results and Discussion

### 3.1. Evaluation of the Sensitivity of STAR and Explorer 2.0 for Control Reference Antibiotics and Salinomycin

Table 1 summarises the sensitivity of the antibiotic standards for STAR and Explorer 2.0. Inhibition zones were created on the *Bacillus subtilis* BGA plate for the streptomycin and ciprofloxacin standards. Sensitivity for tylosin was proven on the *Kocuria rhizophila* ATCC 9341 and *Bacillus stearothermophilus* var. *calidolactis* ATCC 10149 plates. *Bacillus cereus* ATCC 11778 signifies sensitivity for chlortetracycline and *Escherichia coli* ATCC 11303 signifies sensitivity for ciprofloxacin. Positive results for Explorer 2.0 were recorded in the case of salinomycin at concentrations of 100 µg·L^−1^ and 150 µg·L^−1^.

### 3.2. Explorer 2.0

With more than 75% of the antibiotics produced by the pharmaceutical industry being consumed in the livestock sector, the use of antibiotics in the veterinary sector is widespread. Consequently, to prevent the presence of antibiotic residues in food, they must be properly managed regarding their dose or withdrawal period [20,40].

The strategy for current residue control is based on two sequential steps: screening and confirmation. A screening method is used to detect the presence of a substance or class of substances at the level of interest (at or below their MRLs) [2,14,41]. In residue screening, it is best to choose a method that can detect a broad spectrum of antibiotics easily, quickly, and at a low cost. These are screening methods, which are usually microbiologically based. Starting from the logical assumption that most samples are free of residues, these methods lead to contaminated (positive) samples being easily identified from the rest [20,40].

The results of screening for the presence of salinomycin residues in muscle, liver and kidney samples were analysed using Explorer 2.0 (Table 2). Because Explorer 2.0 rated the sample positive if the absorbance value was greater than or equal to fifty-six (≥56), positive results were recorded for C(30) and C(33) samples for all the tissues analysed. Negative results were recorded for C(37), H, F and FH for all the tissues analysed. The measured values decreased constantly in the order of C(30), C(33), C(37), H, F and FH. The lowest values obtained were detected in the FH experimental group for all the tissues analysed.

Zeulab, a leading company in the development of antibiotic testing methods in meat, has designed a unique system that places it at the forefront of screening methods. The Explorer 2.0 and e-Reader system is the most efficient solution for detecting a wide range of antibiotic residues in meat. Using this simple, low-cost, and rapid test, the operator (slaughterhouse) can screen for residues from the main antibiotic groups: beta-lactams, tetracyclines, macrolides, sulphonamides and aminoglycosides. The LOD of the selected antibiotics is determined for the Explorer 2.0 test (Table 3) so that the given values correspond to the valid determination of the MRL values. The test should capture antibiotic residues at the MRL level in order to avoid false positive or false negative results [18,19].

Despite the fact that Explorer 2.0 is not primarily intended for the detection of salinomycin by the manufacturer, it is demonstrably possible to detect salinomycin residues with this microbial inhibition test, as the detected results of the LOD analysis show in Table 1. In this study, the lowest value of salinomycin recorded by the Explorer 2.0 assay was at a concentration of 100 µg·L^−1^, where a positive result determined by the e-Reader was recorded, with an absorbance value reading of 98 (Table 1). Similar values of the LOD were recorded by Kožárová et al. (2020) for the microbial inhibition test Premi^®^ Test based on the same principle of inhibiting the growth of *Bacillus stearothermophilus* var. *Calidolactis* [2].

The investigated samples were collected in the control group of animals on the last day of salinomycin administration: C(30), on the third day of the withdrawal period: C(33) and on the seventh day of the withdrawal period: C(37). In the experimental groups (H, F and FH), tissue samples were collected on the last day of fattening, on the seventh day of the withdrawal period. Explorer 2.0 detected salinomycin residues in the muscle, liver and kidney samples of control groups C(30) and C(33) (Table 2).

### 3.3. STAR

The results of screening for the presence of salinomycin in the muscle, stomach, heart, liver, spleen and kidney tissue samples were analysed using the STAR method for the control group: C(30), C(33) and C(37); and the experimental groups: H, F and FH (Table 4). Inhibition zones (mm) were created on the *Kocuria rhizophila* ATCC 9341 and *Bacillus stearothermophilus* var. *calidolactis* ATCC 10149 plates for tissue samples belonging to the C(30), C(33) and C(37) groups. Zero inhibition zones were created for stomach samples belonging to the C(30) and C(33) groups, and for muscle and stomach samples belonging to the C(37) group. Likewise, zero inhibition zones were created for the H, F and FH groups on the *Kocuria rhizophila* ATCC 9341 and *Bacillus stearothermophilus* var. *calidolactis* ATCC 10149 plates. Zero inhibition zones were created on the *Bacillus subtilis* BGA, *Bacillus cereus* ATCC 11778, and *Escherichia coli* ATCC 11303 plates for all the samples analysed.

Because the STAR rated the sample positive if the inhibition zone was 2 mm and greater on the *Kocuria rhizophila* ATCC 9341 plates, the liver and spleen samples were evaluated as positive in control groups C(30) and C(33). Other tissue samples analysed on the *Kocuria rhizophila* ATCC 9341 plates were evaluated as negative despite the measured diameters, which were below the level of positivity of the inhibition zone. As the STAR rated the sample as positive if the inhibition zone was 4 mm and greater on plates, the muscle, heart, liver, spleen and kidney samples were evaluated as positive in the control groups C(30) and C(33). Other tissue samples analysed on the *Bacillus stearothermophilus* var. *calidolactis* ATCC 10149 plates were evaluated as negative despite the measured diameters, which were below the level of positivity of the inhibition zone.

Despite the fact that the STAR method is not primarily intended for the detection of salinomycin residues, the use of this method for this purpose is validated by determining the sensitivity tosalinomycin in this study, as shown in the Table 1. Sensitivity to salinomycin was confirmed in the case of the test bacterium *Bacillus stearothermophilus* var. *calidolactis* at the lowest concentration of 50 µg·L^−1^ by the formation of an inhibition zone of 2.59 ± 0.18 mm. Similar results were demonstrated in the case of the study byKožárová et al. (2020), proving the suitability of using the *Bacillus stearothermophilus* var. *calidolactis* ATCC 10149 as an adept for salinomycin screening, whether in the form of a microbial inhibition test or the STAR method [2].

### 3.4. ELISA

To determine the type and concentration of the antibiotic in the contaminated sample, specific methods based on immunochemical (ELISAs) or chromatographic (liquid chromatography-mass spectrometry/LC-MS/) techniques should be used. These are the so-called identification or confirmation methods [20].

Immunoassays, coupled with advances in signal amplification, typically offer a high level of sensitivity. Due to the immuno-selection of the analyte of interest, other non-binding analytes are removed and any masking of low-abundance proteins by highly abundant proteins is limited, aiding sensitivity [40,42]. Due to the high selectivity of ELISA, which is bound to not one but a maximum of two analytes, we consider this method to be a highly reliable method that is suitable as a confirmatory analysis in this study.

**Figure 1:**
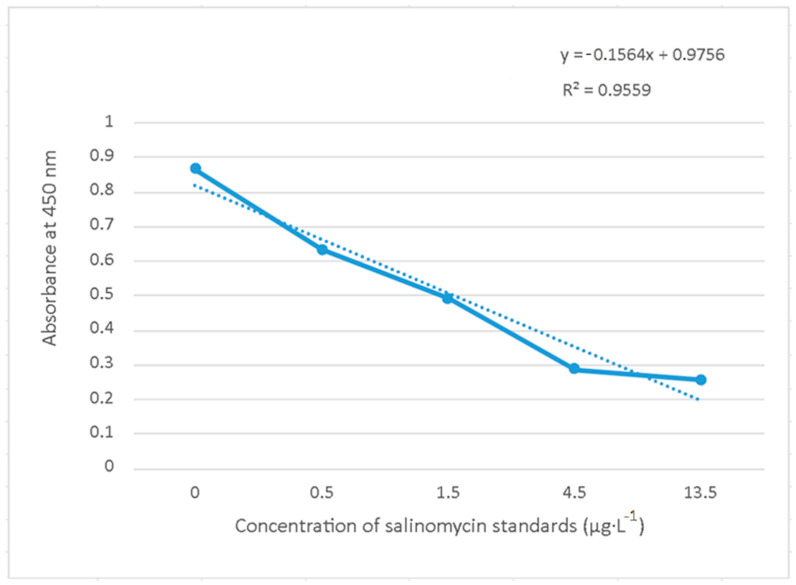
Standard curve of the salinomycin standards in the concentration range of 0–13.5 µg·L^−1^ with the expression of the regression equation and correlation coefficient (R^2^).

The content of salinomycin residues in the chicken muscle samples was analysed quantitatively viacompetitive enzyme immunoassay ELISA using the commercial Salinomycin ELISA Kit. To accurately determine the concentration of tested samples, astandard curve was compiled based on analysis of the standards at the concentrations of 0 µg·L^−1^, 0.5 µg·L^−1^, 1.5 µg·L^−1^, 4.5 µg·L^−1^ and 13.5 µg·L^−1^. Based on the correlation between the absorbance at 450 nm and the concentrations of the salinomycin standards, a standard curve was created with a regression equation (y = −0.1564x + 0.9756) and a correlation coefficient (R^2^ = 0.9559) (Figure 1).

Using ELISA, the chicken muscle samples from the control groups: C(30), C(33) and C(37) and experimental groups: H, F and FH were analysed. Based on four observations, the mean value of absorbance with the expression of standard deviation was calculated for each sample (Table 5, Column 2). Given the absorbance values are in an inverse relationship with the target concentration in the sample, the resulting concentrations of salinomycin in the analysed samples (Table 5, Column 3) were calculated on the basis of the regression equation (y = −0.1564x + 0.9756) obtained during the formation of the standard curve (Figure 1). The highest concentration of salinomycin was detected in the control group in the sample C(30) (4.749 µg·L^−1^), while the lowest concentration of salinomycin was detected in the sample of the experimental group FH (0.310 µg·L^−1^). The content of salinomycin significantly decreased in the order of C(30), C(33), C(37), F, H and FH (*p* < 0.001) (Table 5, Column 3). The highest significant change (*p* < 0.001) was recorded between the C(30) and C(33), C(37), H, F and FH samples; as well as between the C(33) and H, F, FH samples; and C(37) and H, F, FH samples. A lower significant change (*p* < 0.01) occurred between the C(33) and H samples; and the F and FH samples. The evaluated values of the salinomycin content of the C(33) and C(37) samples did not show any significant change (*p* > 0.05).

In this study, the analysis of salinomycin was limited by the detection capabilities of the chosen ELISA detection method. The Salinomycin ELISA Kit works with intra-assay precision of ≤6%, inter -assay precision of ≤10%, in the detection range of 0.5–13.5 µg·L^−1^, and at a sensitivity of 0.5 µg·L^−1^. The LOD of the Salinomycin ELISA Kit is set by the manufacturer for chicken and duck meat at the concentration of 2 µg·L^−1^, for milk at the concentration of 1 µg·L^−1^, and for animal feed at the concentration of 200 µg·L^−1^.

Commission Implementing Regulation (EU) 2017/1914 specifies the MRLs of salinomycin residues in muscle at the concentration of 15 µg·Kg^−1^, inliver at the concentration of 150 µg·Kg^−1^, in kidney at the concentration of 40 µg·Kg^−1^, and inskin/fat at the concentration of 150 µg·Kg^−1^ [4]. The highest value of salinomycin detected at the concentration of 4.749 µg·L^−1^ in the muscle sample came from the control group, taken on the last day of fattening with medicated feed. The detected concentration of salinomycin was well below the established MRL for salinomycin in muscle samples set by this Regulation at the concentration of 15 µg·Kg^−1^ [4].

In the study by Kennedy et al. (1995), the residual concentrations of salinomycin were measured in the tissues of broilers following feeding with medicated feed containing 60 mg·Kg^−1^ of salinomycin. Salinomycin residues were present only at very low concentrations in liver and muscle, and they fell below the limit of decision of the assay within 2 days of withdrawal of the medicated feed [43].

To evaluate the effect of the addition of humic substances and fermented products on the content of salinomycin residues in poultry tissues, available screening methods (Explorer 2.0 and STAR) and confirmatory analysis (ELISA) were used. The results obtained were compared between control groups, which included samples taken on the last day of administration of feed medicated with salinomycin C(30), samples taken on the third day of the withdrawal period C(33) and samples taken on the last day of fattening C(37); and samples from experimental groups H, F and FH taken on the last day of fattening. The results showed the presence of salinomycin residues in the control groups in samples taken on the last day of the administration of medicated feed containing salinomycin and also in samples taken on the third day of the withdrawal period.

As the European Food Safety Authority (EFSA) concluded in Commission Implementing Regulation (EU) 2017/1914 that salinomycin sodium does not have an adverse effect on animal health, human health or the environment and is effective in the control of coccidiosis in chickens for fattening, the exposure estimates at the highest use level indicated an acceptable withdrawal time of zero days [4].

Many beneficial effects of humic substances and fermented products have been monitored in selected parameters and confirmed in many studies [32,33,34,35,37,38,42]. In terms of parameters such as immunostimulation, a positive effect on the intestinal microbiota, influence on fatty acid composition, improvement of the oxidative stability of meat, and stimulating effect on cellular immunity, without a negative effect on haematological and biochemical parameters, were recorded as significant positive effects. From the findings, it is possible to infer the positive impact of these bioactive substances on the health and production indicators of animals, which could have an impact on the quality and safety of animal production products and, ultimately, a positive effect on the health of the consumer.

## 4. Conclusions

Humic substances and fermented products are natural substances with positive effects on animal health and, thus, also on human health. Due to the chemical predispositions of humic substances in synergy with the effect of fermented products added to poultry feed and the potential of reducing the presence of antimicrobial residues in poultry tissues, the aim of this study was to evaluate the effect of the addition of humic substances and fermented products to the feed supplemented with the coccidiostat salinomycin on the content of salinomycin residues in the edible tissues of broiler chickens usingtwo microbial inhibition tests and a competitive enzyme immunoassay. All the methods used provided similar results and showed the positive influence of the administration of humic substances and fermented products on the content of salinomycin residues in chicken tissues. Despite the fact that the salinomycin residues were below the EU maximum residue limit in all the examined samples, the need to control the residues of antimicrobial substances, including coccidiostats, in animal products of food animals is necessary and justified in order to protect human health.

## Figures and Tables

**Table 1:** Sensitivity of control reference antibiotic standards and salinomycin for STAR and Explorer 2.0.

Standard	*Bacillus subtilis* BGA	*Kocuria rhizophila* ATCC 9341	*Bacillus cereus* ATCC 11778	*Escherichia coli* ATCC 11303	*Bacillus stearothermophilus* ATCC 10149	Explorer 2.0
STM	4.75 ± 0.09	0	0	0	0	-
TYL	0	3.88 ± 0.25	0	0	5.39 ± 0.08	-
CHTC	0	0	5.22 ± 0.02	0	0	-
CFC	8.06 ± 0.51	0	0	6.58 ± 0.31	0	-
SD	0	0	0	0	21.54 ± 0.15	-
SAL(50)	0	0	0	0	2.59 ± 0.18	−(53)
SAL(100)	0	0	0	0	5.38 ± 0.14	+(98)
SAL(150)	0	0	0	0	8.32 ± 0.15	+(111)
NC	0	0	0	0	0	−(40)

STAR: All data are the mean ± SD of six observations measured in mm; Explorer 2.0: Numerical values represent the absorbance measured by the e-Reader device; ≥56 = positive, <56 = negative; STM = streptomycin, TYL = tylosin, CHTC = chlortetracycline, CFC = ciprofloxacin, SD = sulphamethazine; SAL(50) = salinomycin standard at the concentration of 50 µg·L^−1^, SAL(100) = salinomycin standard at the concentration of 100 µg·L^−1^, SAL(150) = salinomycin standard at the concentration of 150 µg·L^−1^, NC = negative control.

**Table 2:** Results of the screening of salinomycin residues in chicken tissues using the Explorer 2.0 test with absorbance values determined by e-Reader.

Groups of Experimental Animals						
Sample	C(30)	C(33)	C(37)	H	F	FH
Muscle	+(73)	+(62)	−(50)	−(36)	−(37)	−(19)
Liver	+(80)	+(77)	−(54)	−(39)	−(36)	−(23)
Kidney	+(82)	+(76)	−(51)	−(38)	−(38)	−(22)

Numerical values represent the absorbance measured by the e-Reader device; ≥56 = positive (+), <56 = negative (−).

**Table 3:** Limits of detection of Explorer 2.0 and maximum residue limits for antibiotic residues in muscle, liver and kidney set by European legislation.

Antibiotic	Muscle		Liver		Kidney	
	LOD	MRL	LOD	MRL	LOD	MRL
Amoxicillin	10	50	-	-	≤25	50
Cephalexin	200	200	300	200	250–500	1000
Cloxacillin	100	300	-	-	-	-
Ceftiofur	200	1000	-	-	-	-
Doxycycline	100	100	≤100	300	≤200	600
Erythromycin	300	200	-	-	300	200
Gentamicin	50–100	50	≤100	200	-	-
Lincomycin	-	-	-	-	≤500	1500
Neomycin	200	500	-	-	≤1000	5000
Oxytetracycline	200	100	-	-		
Penicillin G	≤20	50	-	-	-	-
Sulphathiazole	100	100	-	-	≤50	100
Sulphadiazine	100	100	50-100	100	100	100
Sulphamethazine	-	-	-	-	50–100	100
Sulphadimethoxine	100	100	-	-	-	-
Tylosin	100–150	100	≤50	100	≤50	100

LOD = limit of detection; MRL = maximum residue limit. Numerical values are expressed in µg.Kg^−1^.

**Table 4:** Overview of the mean diameters and the standard deviations of the inhibition zones (mm ± SD) recorded using the STAR method for the analysis of salinomycin residues for tissue from the control and experimental groups.

			Bacterial Test Strain			
Groups of Experimental Animals	Tissue	*Bacillus subtilis* BGA	*Kocuria* *rhizophila* ATCC 9341	*Bacillus cereus* ATCC 11778	*Escherichia coli* ATCC 11303	*Bacillus* *stearothermophilus* ATCC 10149
C(30)	Muscle	0	0.59 ± 0.09	0	0	**5.18 ± 0.40**
	Stomach	0	0	0	0	3.43 ± 0.15
	Heart	0	1.43 ± 0.22	0	0	**6.45 ± 0.24**
	Liver	0	**4.32 ± 0.62**	0	0	**7.47 ± 0.86**
	Spleen	0	**3.64 ± 0.09**	0	0	**7.96 ± 0.37**
	Kidney	0	1.01 ± 0.14	0	0	**6.70 ± 0.29**
C(33)	Muscle	0	0.34 ± 0.03	0	0	**4.00 ± 0.27**
	Stomach	0	0	0	0	2.98 ± 0.12
	Heart	0	1.29 ± 0.17	0	0	**4.78 ± 0.34**
	Liver	0	**2.19 ± 0.28**	0	0	**5.54 ± 0.41**
	Spleen	0	**2.49 ± 0.31**	0	0	**5.83 ± 0.26**
	Kidney	0	0.87 ± 0.02	0	0	**4.61 ± 0.15**
C(37)	Muscle	0	0	0	0	2.74 ± 0.32
	Stomach	0	0	0	0	1.96 ± 0.24
	Heart	0	0.43 ± 0.24	0	0	3.07 ± 0.21
	Liver	0	0.97 ± 0.26	0	0	3.38 ± 0.17
	Spleen	0	0.51 ± 0.17	0	0	2.53 ± 0.34
	Kidney	0	0.94 ± 0.19	0	0	3.57 ± 0.28
H	Muscle	0	0	0	0	0
	Stomach	0	0	0	0	0
	Heart	0	0	0	0	0
	Liver	0	0	0	0	0
	Spleen	0	0	0	0	0
	Kidney	0	0	0	0	0
F	Muscle	0	0	0	0	0
	Stomach	0	0	0	0	0
	Heart	0	0	0	0	0
	Liver	0	0	0	0	0
	Spleen	0	0	0	0	0
	Kidney	0	0	0	0	0
FH	Muscle	0	0	0	0	0
	Stomach	0	0	0	0	0
	Heart	0	0	0	0	0
	Liver	0	0	0	0	0
	Spleen	0	0	0	0	0
	Kidney	0	0	0	0	0
	NC	0	0	0	0	0

Bold numerals represent the positive results. All data are the mean ± SD of six observations.

**Table 5:** Means and standard deviations (±SD) of the absorbance and calculated concentrations (µg·L^−1^) of the salinomycin residues in the chicken muscle samples from the control and experimental groups.

Control/Experimental Group	Absorbance at 450 nm (±SD)	Salinomycin Concentration (µg·L^−1^ or µg·Kg^−1^)
C(30)	0.233 ± 0.006 a	4.749
C(33)	0.505 ± 0.006 b	3.008
C(37)	0.584 ± 0.017 c	2.502
H	0.925 ± 0.019 df	0.324
F	0.826 ± 0.011 e	0.953
FH	0.927 ± 0.048 f	0.310

a, b, c, d, e, f = Means within a column different superscript differ (*p* < 0.001). Absorbance data are the mean ± SD of four observations.

## Data Availability

Data is contained within the article.
